# Vacuum Heat Treatment Regulates the Bamboo–Epoxy Interface: Balancing Mechanical Performance and Thermal Stability

**DOI:** 10.3390/polym18202511

**Published:** 2026-10-14

**Authors:** Zilu Liang, Haiyun Jiang, Yimin Tan

**Affiliations:** School of Packaging and Materials Engineering, Hunan University of Technology, Zhuzhou 412007, China

**Keywords:** bamboo/epoxy composites, vacuum heat treatment, interface modification, mechanical properties

## Abstract

The sustainable replacement of conventional synthetic-fiber composites requires renewable reinforcements that combine low density with high load-bearing capability. However, the hydrophilic nature of bamboo restricts effective stress transfer at the bamboo–epoxy interface, while excessive thermal modification can further compromise the structural integrity of the reinforcement. Here, a vacuum-thermal interfacial regulation strategy was employed to establish a favorable balance between interfacial strengthening and thermally induced structural degradation. Moderate treatment improved resin coverage and interfacial contact while preserving the bamboo structure, thereby promoting efficient stress transfer across the bamboo–epoxy interface. Owing to this synergistic interfacial regulation, the composite treated at 160 °C for 4 h achieved tensile and flexural strengths of 174.49 and 219.55 MPa, corresponding to improvements of 49.60% and 29.47%, respectively, over the untreated composite, while excessive treatment caused renewed interfacial defects and mechanical deterioration. Meanwhile, vacuum heat treatment also enhanced thermal stability, increasing T5% from 216.55 to 260.08 °C and the residual mass at 500 °C from 24.18% to 28.28%. These results reveal a processing-window mechanism governed by the competition between interfacial strengthening and thermal degradation, providing an effective route for developing high-performance and sustainable bamboo/epoxy composites.

## 1. Introduction

The increasing demand for lightweight, renewable, and environmentally sustainable materials has accelerated the development of natural-fiber-reinforced polymer composites. Compared with conventional synthetic reinforcements, natural fibers possess advantages such as low density, renewability, low cost, and favorable specific mechanical properties, making them attractive for applications in transportation, construction, and packaging [1,2,3]. Among the various lignocellulosic resources, bamboo is particularly promising because of its rapid growth, high specific strength, and favorable mechanical performance [4,5,6,7]. When combined with polymer matrices, bamboo can provide a renewable load-bearing framework while reducing dependence on conventional inorganic reinforcements. Therefore, improving the structural performance and service stability of bamboo-based polymer composites is of both scientific and practical importance.

A major challenge in developing high-performance bamboo/polymer composites lies in the bamboo–polymer interface. Bamboo is mainly composed of cellulose, hemicellulose, and lignin and contains abundant polar hydroxyl groups together with moisture, starch, sugars, and low-molecular-weight extractives [8,9]. These characteristics give bamboo a strongly hydrophilic surface, whereas polymer matrices such as epoxy exhibit comparatively lower polarity. The resulting interfacial incompatibility can lead to insufficient resin wetting, incomplete interfacial contact, void formation, fiber pull-out, and inefficient stress transfer, thereby limiting the mechanical performance of the composites. In addition, the final composite properties are jointly affected by reinforcement morphology, resin impregnation, porosity, fiber orientation, and curing conditions [10,11]. Consequently, improving the bamboo–epoxy interface without simultaneously damaging the intrinsic bamboo structure remains a key difficulty in the design of high-performance bamboo/epoxy composites.

Various physical and chemical modification strategies have been developed to improve the interfacial compatibility of bamboo-based composites. Alkali treatment has been widely used to modify bamboo surfaces and improve interfacial bonding in bamboo/epoxy systems, while silane coupling agents have been employed to enhance compatibility between bamboo fibers and epoxy matrices [12]. Heat-treated or carbonized bamboo particles have also been introduced into polymer systems to regulate composite properties [13]. In parallel, different molding routes, including hot pressing, compression molding, extrusion, resin transfer molding, and vacuum-assisted resin transfer molding (VARTM), have been applied to bamboo strips, fibers, fabrics, and hybrid reinforcements [14,15,16,17,18]. Although chemical modification can effectively alter bamboo surface characteristics, it generally requires additional reagents and processing steps and may generate chemical waste [19,20,21,22]. Thermal modification provides a comparatively simple alternative and can reduce moisture and thermally labile constituents while changing the surface chemistry and morphology of bamboo. However, conventional thermal modification is commonly performed under atmospheric or liquid-assisted conditions, where oxygen exposure or heat-transfer media may influence the degradation pathway and surface evolution of bamboo. Vacuum heat treatment provides an oxygen-limited environment that suppresses oxidation-related reactions and promotes the removal of moisture and volatile degradation products during heating. This enables the surface and chemical regulation of bamboo without introducing additional chemical reagents or liquid media, providing a distinct strategy for improving bamboo–polymer interfacial compatibility while maintaining structural integrity. Despite these advantages, the effectiveness of vacuum heat treatment remains highly dependent on treatment severity. Excessive temperature or prolonged treatment can promote hemicellulose degradation, drying shrinkage, embrittlement, and microstructural damage, thereby reducing the intrinsic load-bearing capability of bamboo [7,21,23]. Therefore, existing modification methods still face a fundamental trade-off between improving interfacial compatibility and preserving the structural integrity of bamboo.

Based on these considerations, vacuum heat treatment offers a promising route for regulating the bamboo–epoxy interface while minimizing chemical consumption and reducing oxidative degradation during heating. Compared with conventional thermal treatment, vacuum-assisted heat treatment provides a low-oxygen environment that can reduce oxidative reactions and regulate the degradation of thermally sensitive components. Therefore, the application of vacuum conditions may help balance surface modification and structural preservation during thermal treatment [24]. Compared with hydrothermal and oil-heat treatments, vacuum heat treatment avoids the introduction of additional liquid media and can facilitate the removal of moisture and volatile constituents under reduced oxygen exposure [23]. From a materials-design perspective, an appropriate treatment condition is expected to remove part of the moisture and low-molecular-weight surface constituents, improve bamboo surface condition and epoxy infiltration, and strengthen interfacial stress transfer. However, once the treatment intensity exceeds an appropriate range, degradation of thermally sensitive constituents, fiber shrinkage, embrittlement, and microcrack formation may gradually dominate, leading to deterioration of the bamboo skeleton and interfacial integrity. Therefore, the key to vacuum heat-treatment design is not simply to maximize treatment temperature or duration, but to establish a suitable processing window that balances interfacial strengthening and thermally induced structural degradation. VARTM is further employed in this study to promote resin infiltration into continuous bamboo-strip reinforcements and reduce molding-related interfacial defects [16], thereby allowing the influence of vacuum heat treatment on the bamboo–epoxy interface to be more clearly evaluated.

In this work, bamboo strips were subjected to vacuum heat treatment at 140, 160, and 180 °C for 4 and 6 h, followed by the fabrication of bamboo/epoxy composites using VARTM. The effects of heat-treatment temperature and holding time on the mechanical properties, interfacial morphology, and thermal decomposition behavior of the composites were systematically investigated through tensile, flexural, impact, and interlaminar shear tests, scanning electron microscopy (SEM), and thermogravimetric analysis (TGA). Particular attention was paid to clarifying how treatment severity regulates the bamboo–epoxy interface and stress-transfer behavior, and how excessive thermal exposure alters bamboo structural integrity and thermal decomposition characteristics. By correlating interfacial evolution with macroscopic mechanical performance and thermal stability, this study aims to identify an appropriate vacuum heat-treatment window and to reveal the balance between interfacial strengthening and thermal degradation in bamboo/epoxy composites.

## 2. Materials and Methods

### 2.1. Materials

Bamboo strips were mechanically prepared from 3- to 4-year-old Moso bamboo (Phyllostachys edulis) harvested from Guangdong, China. Before vacuum heat treatment, the bamboo strips were conditioned at 23 ± 2 °C and 65 ± 5% relative humidity until reaching moisture equilibrium. The equilibrium moisture content was not quantitatively determined in this study. The outer epidermal layer and inner pith-side layer were removed, and the remaining bamboo was processed into strips with a thickness of approximately 1.7 mm and a width of approximately 9.5 mm. The strips were subsequently arranged and connected using binding threads for composite fabrication. An aerospace-grade epoxy resin system (LT-5093A/LT-5093B-1) supplied by Wells Advanced Materials (Shanghai, China) was used as the matrix. The epoxy resin and curing agent were mixed at a mass ratio of 100:30. The densities of the resin component, curing agent, and mixed uncured system were 1.155, 0.936, and 1.096 g cm^−3^, respectively. After curing, the density of the epoxy system was approximately 1.16 g cm^−3^. The resin exhibited a viscosity of 210 mPa·s at 25 °C and a pot life of approximately 50 min. According to the manufacturer’s recommendation, the epoxy system can be cured at 60 °C for 100 min; in this study, the bamboo/epoxy laminates were cured at 60 °C for 4 h to ensure sufficient resin crosslinking.

### 2.2. Vacuum Heat Treatment of Bamboo Strips

**Figure 1:**
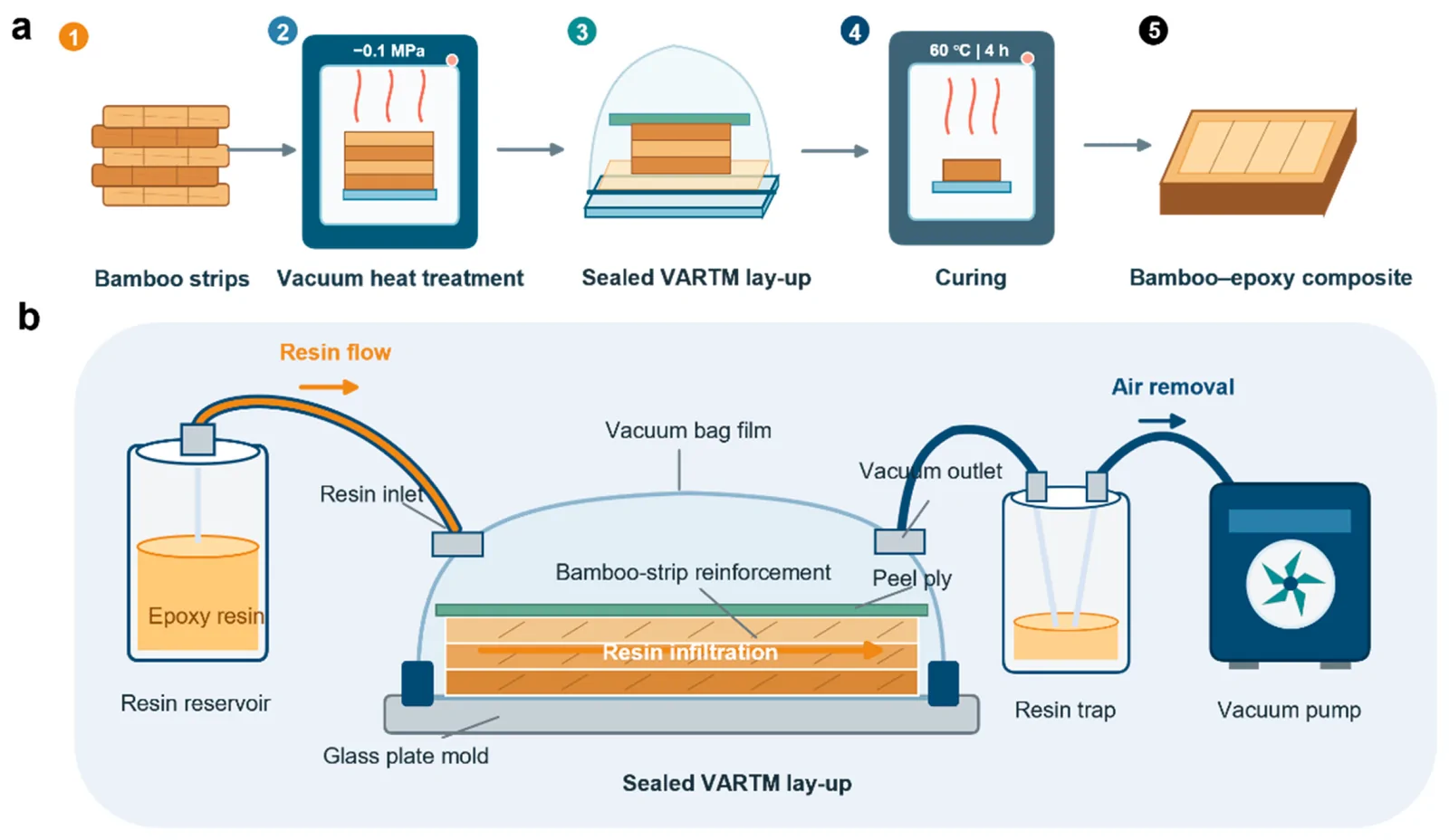
Schematic illustration of the fabrication of bamboo–epoxy composites. (**a**) Overall preparation process involving vacuum heat treatment, VARTM lay-up, curing, and composite formation. (**b**) Detailed VARTM configuration illustrating resin infiltration and vacuum-assisted air removal. In (**a**), gray arrows indicate the fabrication sequence. In (**b**), the orange arrow indicates resin flow, while the blue arrow indicates air removal under vacuum.

The bamboo strips were placed in a vacuum oven and heated under vacuum conditions. The vacuum chamber was evacuated to a gauge pressure of approximately −0.1 MPa, with the experimentally maintained pressure ranging from −0.095 to −0.100 MPa during the treatment process. The temperature was increased from room temperature to the designated treatment temperature within approximately 1 h and subsequently maintained at 140, 160, or 180 °C for either 4 or 6 h. After heat treatment, the samples were allowed to cool naturally to room temperature inside the vacuum oven before removal (Figure 1a). Untreated bamboo strips were used as the control. The resulting groups were designated as Untreated, 140-4, 140-6, 160-4, 160-6, 180-4, and 180-6 according to the treatment temperature and holding time.

### 2.3. Preparation of Bamboo/Epoxy Composites

Bamboo/epoxy composites were fabricated using the vacuum-assisted resin transfer molding (VARTM) process, as illustrated in Figure 1b. A glass plate with dimensions of 2 m × 1 m was used as the mold, and a 55NC release agent (Henkel, Düsseldorf, Germany) was applied to the mold surface before lay-up. Three bamboo strips were aligned longitudinally in the same direction and assembled into a three-layer laminate architecture before epoxy infusion. The VARTM process was performed under a vacuum gauge pressure of approximately −0.1 MPa. The mixed epoxy resin was introduced into the bamboo preform and allowed to flow through the laminate for approximately 20 min to achieve sufficient impregnation. The final laminate thickness was approximately 4.8–5.1 mm. After infusion, the laminates were heated from room temperature to 60 °C within approximately 60 min and cured at 60 °C for 4 h without additional post-curing. The apparent bamboo reinforcement volume ratio was estimated based on the volume of bamboo strips before resin infusion and the total volume of the fabricated laminates. The calculated value ranged from approximately 81% to 87% among the different treatment groups, indicating that the relative bamboo-strip content remained consistent. It should be noted that this value represents the geometric volume ratio of continuous bamboo strips rather than the conventional fiber volume fraction used for separated fiber-reinforced polymer composites. The epoxy resin was introduced through vacuum-assisted infusion, allowing penetration into accessible bamboo structures and improving interfacial bonding between adjacent bamboo strips.

### 2.4. Mechanical Characterization

The mechanical properties of the bamboo/epoxy composites were evaluated by tensile, flexural, interlaminar shear, and impact tests. Tensile tests were performed in accordance with ASTM D3039 [25] using an EM6.105 electronic universal testing machine (TSMT, Shenzhen, China) at a crosshead speed of 1 mm/min. An external extensometer (Sanjing, YB50/5-N, Changchun Sanjing Test Instrument Co., Ltd., Changchun, China) was used to record tensile deformation for the calculation of tensile strain and modulus. The specimens had dimensions of 250 mm × 25 mm × 5 mm. Flexural properties were measured according to ASTM D790 [26] using a CMT5105 universal testing machine (Shenzhen SANS Testing Machine Co., Ltd., Shenzhen, China). Specimens with dimensions of 103 mm × 12 mm × 5 mm were tested at a crosshead speed of 2 mm/min with a support span of 78 mm. Interlaminar shear strength was determined according to ASTM D2344-22 [27] using an EM6.105 electronic universal testing machine with a load capacity of 100 kN and an accuracy class of 0.5 in accordance with ISO 7500-1:2018 [28]. Specimens with dimensions of 33 mm × 10 mm × 5 mm were tested at a crosshead speed of 1.0 mm/min. Impact properties were evaluated according to ISO 179-1:2023 [29] using a SANS Charpy pendulum impact tester (Shenzhen SANS Testing Machine Co., Ltd., Shenzhen, China) at an impact velocity of 2.9 m/s. The impact specimens had dimensions of 60 mm × 10 mm × 5 mm. Statistical analysis was performed using one-way analysis of variance (ANOVA) followed by Tukey’s multiple comparison test to determine the significance of differences among different treatment conditions. Differences were considered statistically significant at *p* < 0.05.

### 2.5. Microstructure

The fracture morphologies and bamboo–epoxy interfacial characteristics of the composites were examined using an SEM (Apreo 2S HiVac, Thermo Fisher Scientific Brno s.r.o., Brno, Czech Republic). Fracture surfaces obtained after mechanical testing were selected for observation to evaluate resin coverage, interfacial gaps, fiber pull-out, fiber fracture, and other characteristic failure features associated with different vacuum heat-treatment conditions.

### 2.6. Thermogravimetric Analysis

Thermogravimetric analysis (TGA) was performed under a nitrogen atmosphere to evaluate the thermal decomposition behavior of the bamboo/epoxy composites. Approximately 80 mg of sample was placed in a platinum crucible and heated from 50 to 600 °C at a constant heating rate of 5 °C/min under a nitrogen flow rate of 25 mL/min. One measurement was performed for each treatment condition, and the reported TG and DTG curves represent individual measurements rather than averaged data. These curves were used to compare the overall thermal degradation behavior of different samples. The temperature corresponding to 5% mass loss (T5%) was used to characterize the onset thermal stability, while the residual mass fraction at 500 °C was used to evaluate the high-temperature residue retention behavior of the composites. Derivative thermogravimetric (DTG) curves were obtained from the corresponding TG data using an identical data-processing procedure for all experimental groups.

## 3. Results and Discussion

### 3.1. Effect of Vacuum Heat Treatment on Mechanical Properties of Bamboo/Epoxy Composites

**Figure 2:**
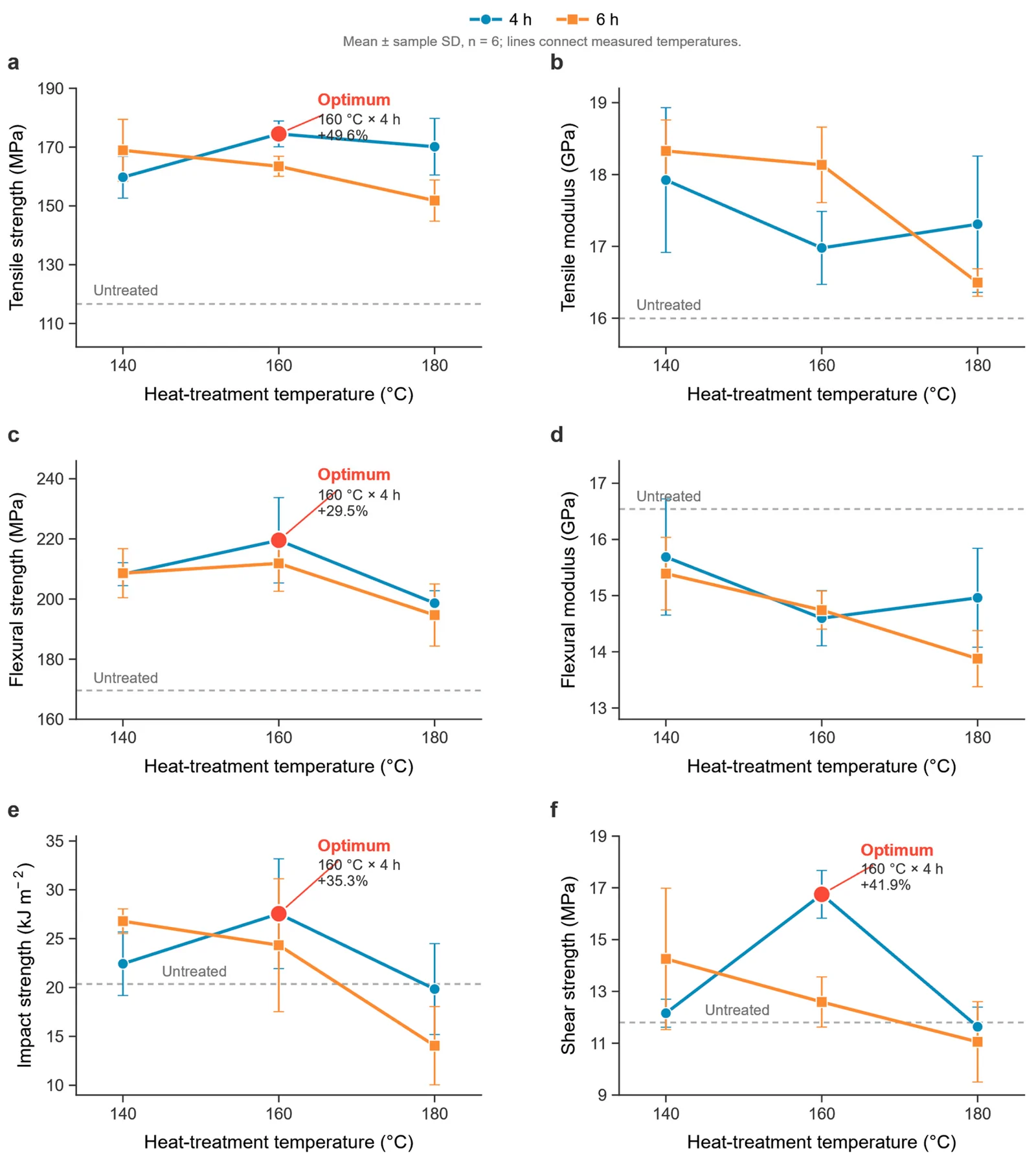
Mechanical properties of the bamboo/epoxy composites under different vacuum heat-treatment conditions: (**a**) tensile strength, (**b**) tensile modulus, (**c**) flexural strength, (**d**) flexural modulus, (**e**) impact strength, and (**f**) interlaminar shear strength. The dashed lines indicate the corresponding values of the untreated composite.

The mechanical properties of bamboo/epoxy composites exhibited a pronounced dependence on both heat-treatment temperature and holding time (Figure 2). In general, moderate vacuum heat treatment enhanced the strength-related properties of the composites, whereas excessive treatment progressively weakened this beneficial effect. In particular, tensile strength, flexural strength, impact strength, and interlaminar shear strength all showed an evident optimum within the investigated treatment window, while the corresponding modulus responses were not fully consistent with the strength evolution. These differences indicate that vacuum heat treatment simultaneously affects the bamboo–epoxy interface and the intrinsic deformation behavior of the bamboo reinforcement.

As shown in Figure 2a, the tensile strength increased substantially after vacuum heat treatment. The untreated composite exhibited a tensile strength of 116.64 MPa, whereas treatment at 140 °C increased the strength to 159.76 MPa after 4 h and 168.91 MPa after 6 h. The maximum value of 174.49 MPa was obtained at 160 °C for 4 h, corresponding to an increase of 49.60% relative to the untreated composite. Notably, the influence of holding time depended strongly on treatment temperature. At 140 °C, extending the treatment from 4 to 6 h further increased the tensile strength, whereas at 160 and 180 °C, prolonged treatment caused a reduction in strength. At 180 °C, the tensile strength decreased from 170.13 MPa after 4 h to 151.81 MPa after 6 h. This temperature–time dependence suggests that moderate thermal exposure favors interfacial regulation, whereas excessive treatment increasingly compromises the integrity of the bamboo reinforcement [11,30]. Statistical analysis was further performed to evaluate the influence of vacuum heat-treatment conditions on tensile strength. The Levene’s test indicated homogeneous variances among groups (*p* = 0.402), and one-way ANOVA confirmed significant differences among treatment conditions (F(6,35) = 39.17, *p* = 4.11 × 10^−14^). Tukey’s HSD analysis showed that the tensile strength of the 160 °C × 4 h composite was significantly higher than those of the untreated, 140 °C × 4 h, and 180 °C × 6 h composites, with adjusted *p* values of 1.24 × 10^−13^, 0.0316, and 0.000218, respectively. However, no significant differences were found between the 160 °C × 4 h composite and the 140 °C × 6 h, 160 °C × 6 h, or 180 °C × 4 h composites, with adjusted *p* values of 0.866, 0.197, and 0.955, respectively. Therefore, the 160 °C × 4 h condition should not be interpreted as uniquely statistically superior to all other heat-treated conditions. Rather, it is identified as the preferred processing condition within the investigated range because it yielded the highest observed mean tensile, flexural, impact, and interlaminar shear strengths, representing the most favorable overall strength-related performance. In contrast, the tensile modulus showed a different response (Figure 2b). Although all heat-treated specimens retained a modulus above that of the untreated composite (16.00 GPa), the maximum modulus of 18.33 GPa was obtained at 140 °C for 6 h rather than at the condition giving the highest tensile strength. This decoupling between strength and stiffness indicates that the improvement in tensile load-bearing capacity cannot be attributed solely to an increase in the intrinsic stiffness of the reinforcement, but is also strongly associated with changes in interfacial stress transfer and structural integrity [31].

A similar treatment-dependent response was observed for the flexural properties (Figure 2c,d). The untreated composite exhibited a flexural strength of 169.58 MPa. After heat treatment at 140 °C, the flexural strength increased to approximately 208 MPa, and the maximum value of 219.55 MPa was again obtained at 160 °C for 4 h, representing an increase of 29.47%. Prolonging the treatment to 6 h at 160 °C reduced the strength to 211.86 MPa, while further increasing the temperature to 180 °C led to a continued decline to 198.63 MPa and 194.66 MPa for 4 and 6 h, respectively. Thus, the flexural-strength response closely followed the tensile-strength trend and further confirmed the existence of an optimum treatment severity [31,32,33]. However, the flexural modulus exhibited the opposite tendency (Figure 2d). Compared with the untreated composite (16.54 GPa), the flexural modulus decreased by 5.14%, 6.95%, 11.73%, 10.88%, 9.58%, and 16.08% after treatment at 140 °C × 4 h, 140 °C × 6 h, 160 °C × 4 h, 160 °C × 6 h, 180 °C × 4 h, and 180 °C × 6 h, respectively. This result indicates that the increase in flexural strength is not accompanied by a corresponding increase in structural stiffness. The different evolution trends between flexural strength and modulus can be attributed to their distinct dependence on composite structure. Flexural strength is strongly affected by interfacial bonding and stress-transfer efficiency, allowing improved resin infiltration and interface contact after moderate vacuum heat treatment to enhance the failure resistance of the composite. In contrast, flexural modulus is mainly governed by the intrinsic stiffness of the reinforcement, fiber architecture, and overall deformation constraint. The reduction in modulus after heat treatment suggests that thermal exposure may reduce the deformation resistance of the bamboo skeleton through structural changes, such as cell-wall modification and dimensional shrinkage, which cannot be fully compensated by improved interfacial adhesion. Therefore, moderate heat treatment enhances load-bearing capacity by improving stress transfer, while the stiffness response remains limited by changes in the intrinsic deformation behavior of the bamboo reinforcement [11,33,34,35].

It should be noted that the relatively large standard deviations observed for tensile and flexural modulus are associated with the intrinsic heterogeneity of natural bamboo reinforcement (Table 1). Bamboo possesses hierarchical anisotropic structures with variations in vascular bundle distribution, fiber bundle density, and cell wall characteristics, which can result in specimen-to-specimen differences in elastic response. Moreover, the modulus of bamboo/epoxy laminates is simultaneously affected by resin impregnation, interfacial bonding, and local structural defects. Therefore, the observed dispersion reflects the combined influence of biological variability and composite structural heterogeneity. Despite these variations, the overall modulus evolution follows the expected trend with treatment severity, where moderate vacuum heat treatment promotes interfacial stress transfer while excessive treatment causes structural deterioration [12].

The impact response provides further evidence that the mechanical effect of heat treatment cannot be described solely in terms of interfacial bonding strength (Figure 2e). Moderate treatment increased the impact strength relative to the untreated composite, with the maximum improvement of approximately 35.3% obtained at 160 °C for 4 h. In contrast, prolonged treatment at 180 °C caused a pronounced reduction in impact resistance. Unlike quasi-static tensile and flexural loading, impact fracture involves multiple energy-dissipation mechanisms, including matrix cracking, interfacial debonding, fiber pull-out, and fiber fracture. Therefore, excessively strong or excessively weak interfacial bonding does not necessarily maximize impact resistance; an appropriate balance between interfacial stress transfer and energy-dissipative failure mechanisms is required [22,36,37,38]. At high treatment severity, thermal degradation and embrittlement of the bamboo reinforcement can reduce its ability to dissipate impact energy, resulting in increasingly brittle failure [13,39].

Interlaminar shear strength, which is particularly sensitive to the reinforcement–matrix interface, further supports the above interpretation (Figure 2f). Vacuum heat treatment initially enhanced the shear strength, and the highest value was again obtained at 160 °C for 4 h, corresponding to an improvement of approximately 41.9% relative to the untreated composite. However, extending the holding time or increasing the treatment temperature beyond this condition caused a marked decrease in shear strength. The close correspondence between the evolution of interlaminar shear strength and the tensile/flexural strength suggests that the mechanical enhancement induced by moderate heat treatment is closely related to improved interfacial load transfer. With excessive thermal exposure, however, degradation and embrittlement of the bamboo structure progressively offset the interfacial benefits and eventually lead to reduced shear resistance [21].

**Figure 3:**
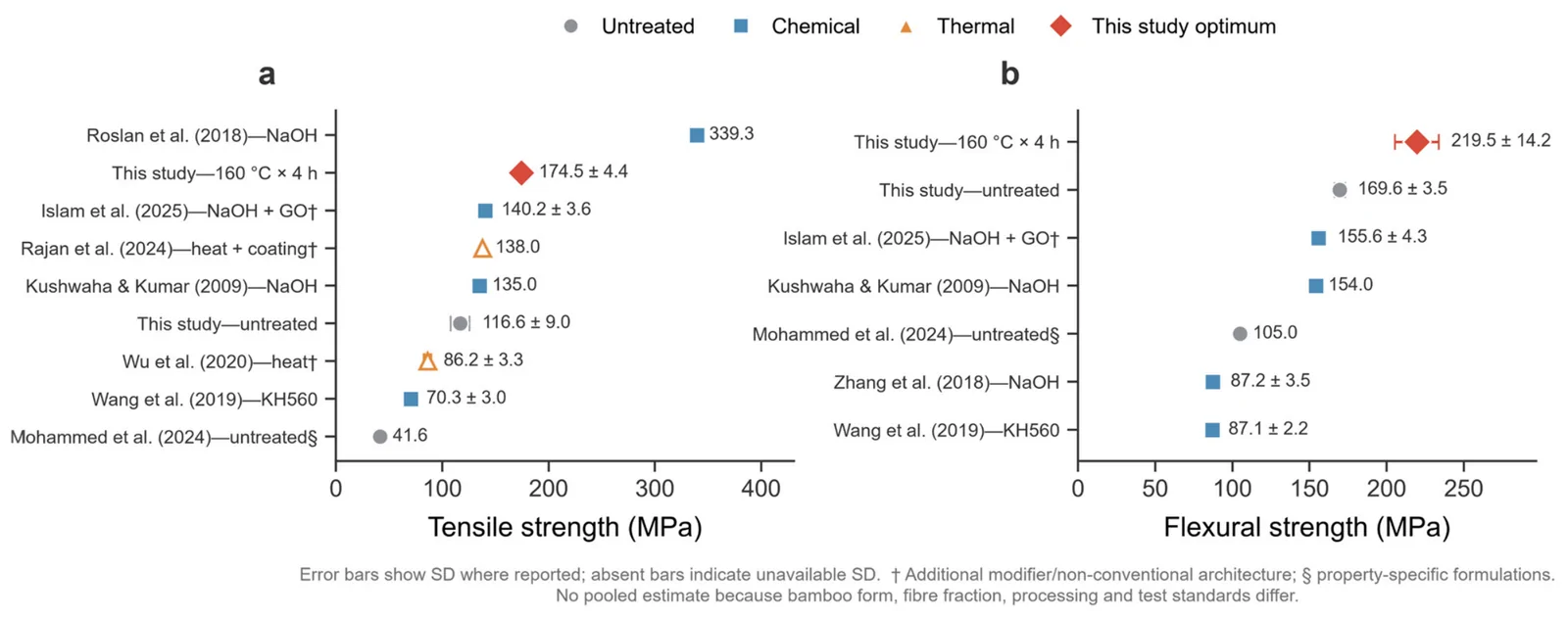
Comparison of the tensile (**a**) and flexural (**b**) strengths of bamboo/epoxy composites with different modification strategies reported in the literature. Error bars represent standard deviations where available.

Taken together, the results in Figure 2 demonstrate that vacuum heat treatment produces a clear processing-window effect rather than a monotonic improvement in mechanical properties. Among the investigated conditions, 160 °C for 4 h exhibited the most favorable strength-related mechanical performance, and the statistical analysis further supported the significance of the treatment effect. In contrast, the maximum tensile modulus occurred at 140 °C for 6 h, and the flexural modulus decreased after heat treatment, confirming that strength and stiffness respond differently to thermal modification. These results imply that the mechanical behavior of bamboo/epoxy composites is governed by the competition between interfacial improvement and thermally induced deterioration of the bamboo reinforcement. To further evaluate the effectiveness of vacuum heat treatment, the tensile and flexural strengths of the optimized bamboo/epoxy composite (160 °C × 4 h) were compared with previously reported untreated, chemically modified, and thermally treated bamboo/epoxy composites (Figure 3). The results indicate that the proposed vacuum heat-treatment strategy achieves competitive mechanical performance without introducing additional chemical reagents, providing an effective balance between interfacial enhancement and processing simplicity.

### 3.2. Interfacial Morphology and Failure Behavior of Bamboo/Epoxy Composites

**Figure 4:**
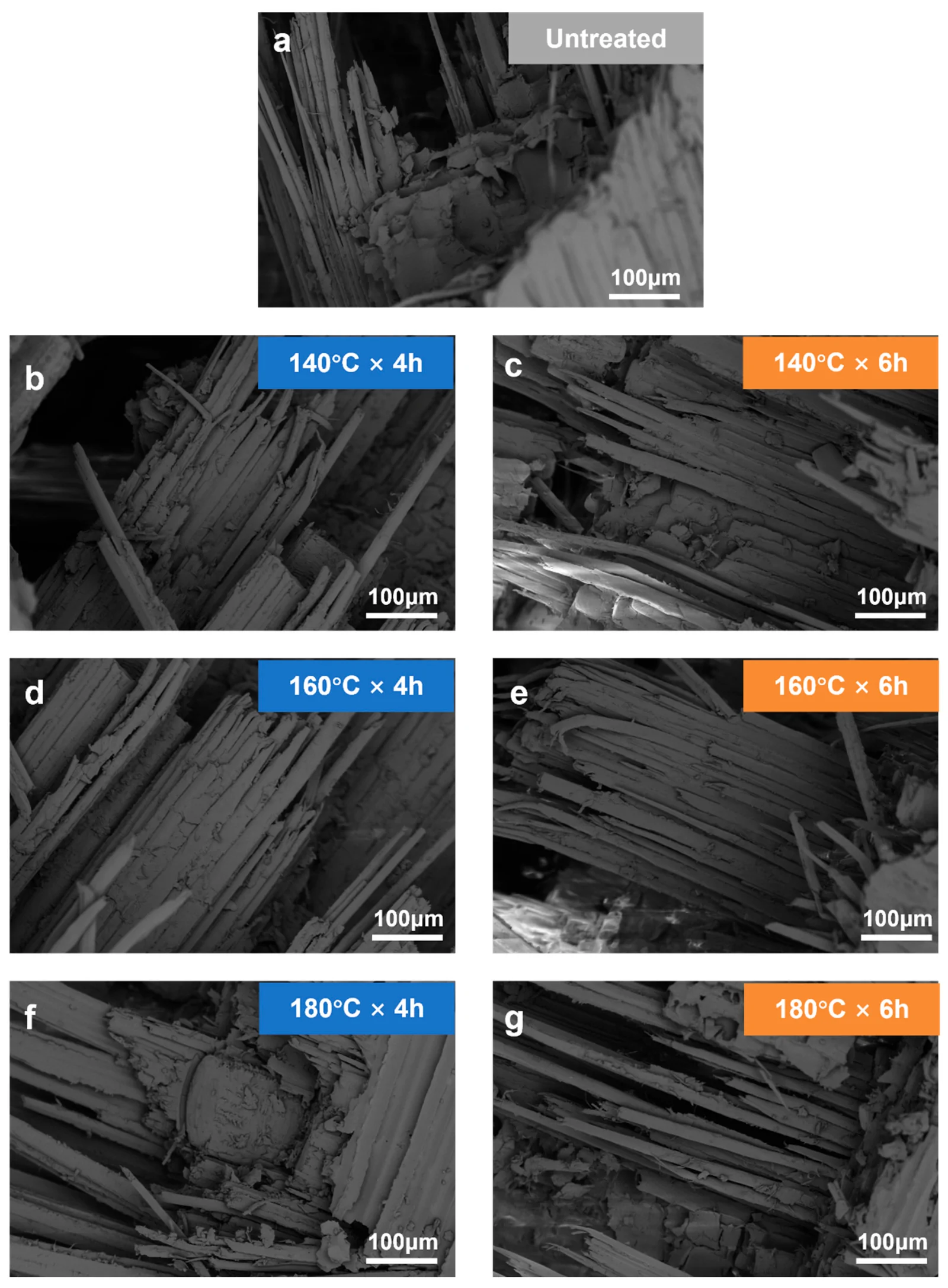
SEM images of the fracture surfaces of the bamboo/epoxy composites under different vacuum heat-treatment conditions: (**a**) Untreated, (**b**) 140 °C × 4 h, (**c**) 140 °C × 6 h, (**d**) 160 °C × 4 h, (**e**) 160 °C × 6 h, (**f**) 180 °C × 4 h, and (**g**) 180 °C × 6 h.

The fracture morphology of the bamboo/epoxy composites exhibited a clear evolution with increasing vacuum heat-treatment severity (Figure 4). In general, the interface changed from relatively poor interfacial contact in the untreated composite to a more compact and integrated morphology after moderate heat treatment, followed by renewed interfacial deterioration under excessive treatment. The untreated composite (Figure 4a) exhibited obvious interfacial gaps, discontinuous resin coverage, exposed fiber bundles, and pronounced fiber pull-out, indicating insufficient resin infiltration and weak interfacial stress transfer. These features indicate that fracture preferentially propagated along the bamboo–epoxy interface, resulting in inefficient stress transfer between the matrix and the reinforcement [40]. This interfacial morphology is consistent with the relatively low tensile, flexural, impact, and interlaminar shear properties observed for the untreated composite in Figure 2.

After heat treatment at 140 °C, the interfacial morphology became noticeably more compact (Figure 4b,c). Compared with the untreated composite, both the 4 h and 6 h samples exhibited reduced interfacial voids and improved resin attachment around the bamboo fibers, although local fiber pull-out and discontinuous interfacial regions were still present. Increasing the treatment temperature to 160 °C further improved the interfacial state. In particular, the 160 °C × 4 h composite (Figure 4d) exhibited more continuous resin coverage, fewer large interfacial gaps, and a more integrated fracture surface, with reduced fiber pull-out compared with the untreated composite. These features suggest enhanced interfacial contact and more effective stress transfer between the bamboo reinforcement and epoxy matrix [41,42]. This condition corresponds closely to the maximum tensile strength, flexural strength, impact strength, and interlaminar shear strength observed in Figure 2, suggesting that the mechanical optimum at 160 °C × 4 h is closely associated with improved interfacial contact and more efficient stress transfer.

When the holding time at 160 °C was extended from 4 to 6 h, the fracture morphology began to change (Figure 4e). More exposed fiber bundles and local interfacial separation could be observed, indicating that prolonged thermal exposure had started to offset the interfacial benefits obtained at the shorter holding time. This tendency became more evident at 180 °C (Figure 4f,g). Although the 180 °C × 4 h sample still retained partial resin adhesion, the fracture surface became more heterogeneous, with increased fiber exposure and local structural defects. After 6 h of treatment, the interface showed more pronounced fiber separation, debonding, and disruption of the bamboo structure. These features are consistent with increasing thermally induced structural damage rather than continued interfacial improvement. Importantly, the SEM observations alone do not directly identify specific chemical degradation reactions; instead, they provide morphological evidence that excessive temperature and prolonged holding time progressively compromise both the bamboo structural integrity and the continuity of the bamboo–epoxy interface.

The evolution of fracture morphology therefore agrees well with the mechanical response summarized in Figure 2. For the untreated composite, insufficient interfacial contact promotes debonding and fiber pull-out, limiting stress transfer. Moderate vacuum heat treatment progressively improves interfacial contact, with the most favorable morphology observed at 160 °C × 4 h, corresponding to the best overall strength-related mechanical performance. With further increases in treatment severity, however, structural damage and renewed interfacial defects become increasingly evident, leading to the decline in mechanical performance observed for the prolonged 160 °C treatment and the 180 °C-treated composites. These results indicate that the mechanical optimum originates from a balance between interfacial improvement and the preservation of bamboo structural integrity. It should be noted that additional higher-magnification SEM images were not available in the present study. Therefore, the current SEM observations mainly characterize the overall fracture morphology and interfacial features, while more detailed identification of local resin adhesion, interfacial gaps, fiber pull-out, and microcracks will require higher-magnification imaging in future work.

### 3.3. Thermal Decomposition Behavior and Thermal Stability of Bamboo/Epoxy Composites

**Figure 5:**
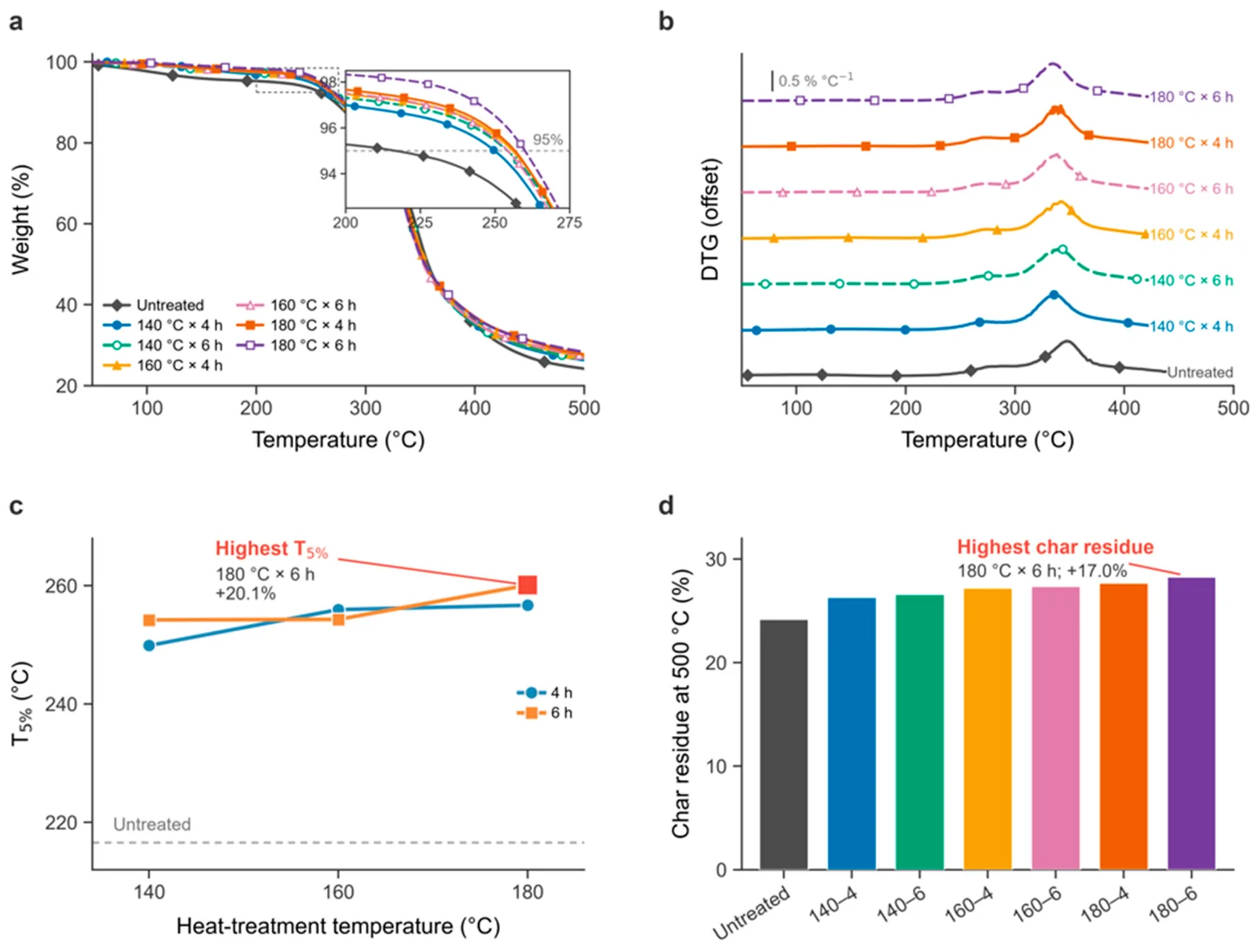
Thermal decomposition characteristics of the bamboo/epoxy composites under different vacuum heat-treatment conditions: (**a**) TG curves with an enlarged view of the initial decomposition region, (**b**) DTG curves, (**c**) 5% mass-loss temperature (T5%), and (**d**) residual mass at 500 °C.

All bamboo/epoxy composites exhibited similar overall TG profiles, indicating that vacuum heat treatment did not substantially alter the principal thermal decomposition pattern of the composites. Clear differences were nevertheless observed in the initial mass-loss region, where the heat-treated samples generally showed delayed early-stage degradation relative to the untreated composite. The enlarged region further highlights the separation among the TG curves during this stage, while a higher residual mass was retained by the heat-treated composites at elevated temperatures (Figure 5a). The corresponding DTG curves revealed that the principal decomposition peaks remained within a relatively similar temperature range, and no monotonic shift toward higher temperatures occurred with increasing treatment temperature or holding time (Figure 5b). These results indicate that vacuum heat treatment mainly affects the onset of thermal degradation rather than uniformly shifting the entire decomposition process toward higher temperatures.

The improvement in initial thermal stability was further reflected by T5%. The untreated composite exhibited a T5% of 216.55 °C, whereas all heat-treated samples showed markedly higher values. At 140 °C, T5% increased to 249.88 °C after 4 h and 254.20 °C after 6 h. Treatment at 160 °C for 4 h further increased T5% to 255.95 °C, while prolonging the holding time to 6 h resulted in a slight decrease to 254.29 °C. At 180 °C, T5% reached 256.68 °C after 4 h and a maximum of 260.08 °C after 6 h, corresponding to an increase of 43.53 °C, or 20.10%, relative to the untreated composite (Figure 5c). This overall increase suggests that vacuum heat treatment reduces the contribution of thermally labile fractions to early-stage decomposition, although prolonged treatment may introduce competing degradation effects under certain conditions [12,13].

A similar enhancement was observed in the residual mass at 500 °C. The untreated composite retained 24.18% of its initial mass, whereas the residual mass increased to 26.30% and 26.59% after treatment at 140 °C for 4 and 6 h, respectively. Further increases were obtained at 160 °C (27.20–27.37%) and 180 °C (27.67–28.28%), with the highest value of 28.28% obtained after treatment at 180 °C for 6 h, representing a 16.96% increase relative to the untreated composite (Figure 5d). The progressive increase in residual mass indicates differences in residue evolution during high-temperature decomposition. This behavior may be influenced by prior volatilization and constituent transformation during vacuum heat treatment, as well as possible changes in the relative proportions of bamboo- and epoxy-derived components in the composite specimens. Therefore, the higher residual mass should be interpreted as a change in high-temperature residue retention. Overall, vacuum heat treatment mainly enhanced the onset thermal stability and altered the high-temperature residue evolution behavior of the bamboo/epoxy composites, while exerting a comparatively limited influence on the temperature range of the principal decomposition stage.

### 3.4. Mechanism of Vacuum Heat Treatment on the Bamboo–Epoxy Interface and Composite Performance

**Figure 6:**
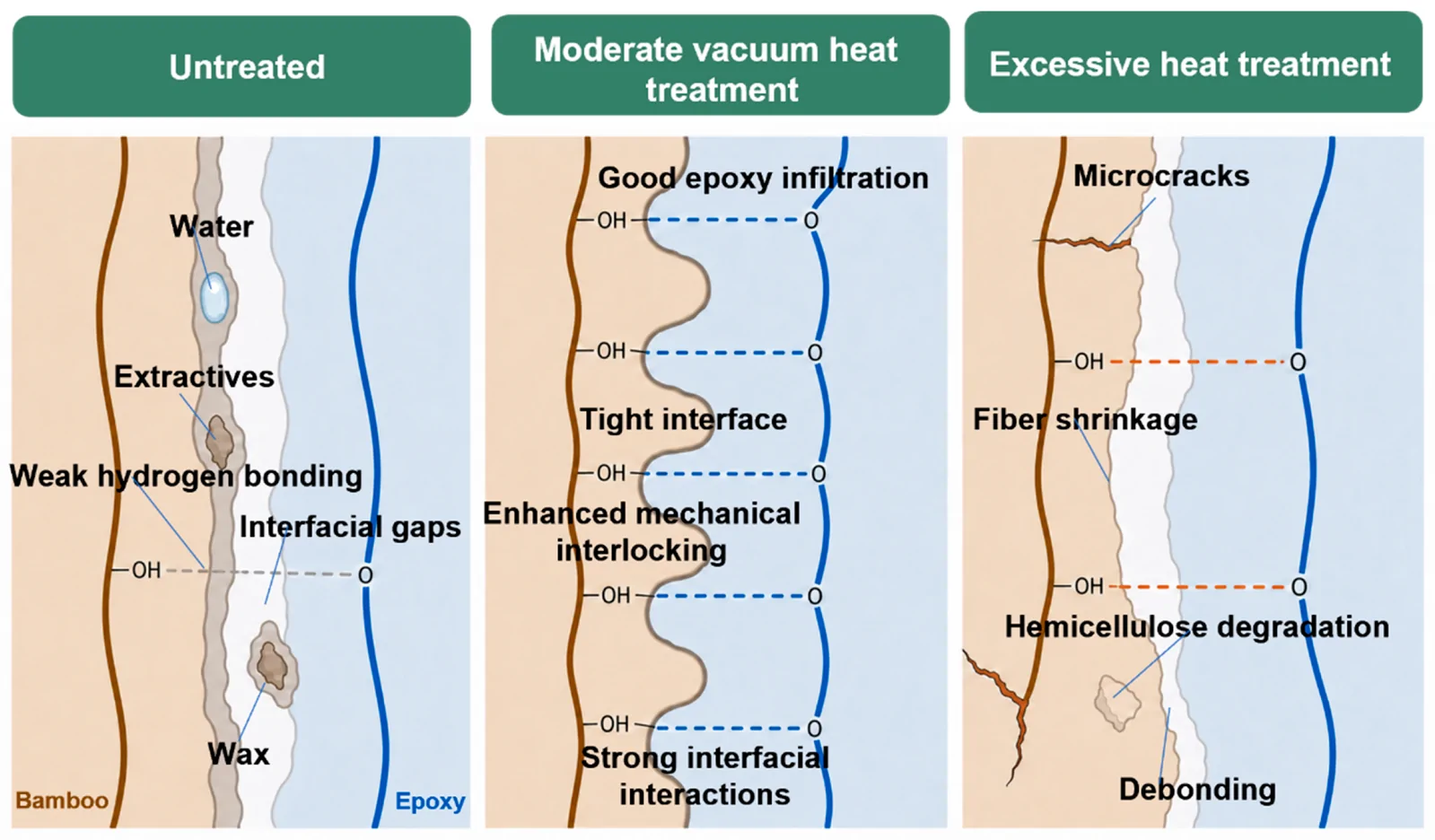
Schematic illustration of the interfacial evolution of the bamboo/epoxy composites under different vacuum heat-treatment conditions: untreated, moderate treatment at 160 °C for 4 h, and excessive treatment at 180 °C for 6 h, highlighting the competition between interfacial strengthening and thermally induced structural degradation. The proposed chemical changes represent possible mechanisms associated with vacuum heat treatment based on previous studies.

The evolution of composite performance with increasing heat-treatment severity can be understood as the result of competing interfacial strengthening and thermally induced structural degradation. The corresponding mechanism is illustrated in Figure 6. The untreated bamboo/epoxy composite exhibited limited interfacial contact, as evidenced by the interfacial gaps, discontinuous resin coverage, and pronounced fiber pull-out observed in the fracture surface. Such an interface restricts effective stress transfer from the epoxy matrix to the bamboo reinforcement and consequently limits the load-bearing capability of the composite. Moderate vacuum heat treatment progressively altered this interfacial state. The removal or transformation of moisture and thermally labile low-molecular-weight constituents, together with changes in bamboo surface morphology, may contribute to improved resin contact and promote more effective mechanical interlocking at the bamboo–epoxy interface [11,30]. Under the 160 °C × 4 h condition, these interfacial benefits were achieved while the structural integrity of the bamboo reinforcement was largely preserved, resulting in a more compact interface and more efficient stress transfer. This balance is consistent with the highest tensile strength, flexural strength, and interlaminar shear strength obtained under this treatment condition.

With further increases in treatment severity, however, the beneficial effect of interfacial modification was progressively offset by thermally induced deterioration of the bamboo structure. The increasing mass loss of bamboo strips after vacuum heat treatment further confirms the enhanced treatment severity. The mass reduction increased from 5.80% at 140 °C × 4 h to 26.25% at 180 °C × 6 h, suggesting progressively intensified removal of volatile components and thermal degradation with increasing temperature and treatment duration (Table 2). Excessive mass loss may accelerate structural deterioration, which contributes to the reduction in mechanical performance under severe treatment conditions. Extending the holding time to 6 h at 160 °C already resulted in increased fiber exposure and local interfacial separation, while treatment at 180 °C produced more pronounced fiber disruption, debonding, and structural defects. These changes indicate that prolonged thermal exposure increasingly compromises reinforcement integrity and interfacial continuity. Consequently, the governing mechanism changes from interfacial improvement to thermal-damage-dominated behavior. In this regime, further removal of surface constituents no longer translates into improved mechanical performance because the loss of bamboo structural integrity and the reappearance of interfacial defects limit effective load transfer. The evolution can therefore be understood as a competition between interfacial strengthening and thermally induced structural degradation: the former dominates under moderate treatment, whereas the latter becomes increasingly important under excessive treatment.

The thermal response follows a different tendency from the mechanical response. Increasing heat-treatment severity continued to increase T5% and the residual mass at 500 °C, with the highest values obtained after treatment at 180 °C for 6 h, even though the mechanical properties had already declined under this condition. This difference indicates that the structural changes detrimental to room-temperature load-bearing performance do not necessarily reduce thermal stability. Prior thermal exposure can remove or transform part of the less thermally stable fractions before subsequent decomposition, thereby delaying early-stage mass loss and contributing to higher residue retention at elevated temperatures [12]. Thus, the effects of vacuum heat treatment on bamboo/epoxy composites involve two competing but not fully synchronized responses: preservation of interfacial and reinforcement integrity governs mechanical performance, whereas the progressive transformation of thermally labile constituents contributes to improved onset thermal stability and higher residual mass retention at elevated temperatures. Among the investigated conditions, 160 °C × 4 h therefore provides the most favorable balance between interfacial strengthening and the preservation of bamboo structural integrity, whereas more severe treatment further improves thermal stabilization at the expense of mechanical integrity.

## 4. Conclusions

This study establishes a vacuum-thermal interfacial regulation strategy for bamboo/epoxy composites by balancing interfacial strengthening with thermally induced structural degradation. Moderate vacuum heat treatment progressively improves resin coverage, interfacial contact, and stress-transfer efficiency while preserving the integrity of the bamboo reinforcement, thereby generating a distinct mechanical processing window. Under the condition of 160 °C for 4 h, the composite achieved tensile and flexural strengths of 174.49 and 219.55 MPa, corresponding to increases of 49.60% and 29.47%, respectively, over the untreated composite, together with the highest interlaminar shear strength. In contrast, excessive thermal exposure induced fiber disruption, interfacial separation, and structural defects, causing the mechanical benefits of interface optimization to diminish. Meanwhile, increasing treatment severity continuously enhances thermal stabilization, with the 180 °C × 6 h composite reaching a T5% of 260.08 °C and a residual mass of 28.28% at 500 °C, mainly through delayed early-stage degradation and increased residual mass retention. The resulting performance evolution therefore arises from the competition between interfacial strengthening and thermal degradation, which decouples the mechanical optimum from the thermal-stability optimum. This processing-window regulation provides a simple and reagent-free route for tailoring the interface and performance of sustainable bamboo/epoxy composites, where the identified 160 °C × 4 h condition offers a practical balance between enhanced interfacial performance and preserved bamboo structural integrity for lightweight structural applications. Nevertheless, the present study primarily focused on the relationship between vacuum heat-treatment conditions, interfacial evolution, and composite performance, while the detailed chemical evolution of bamboo constituents during treatment remains to be further clarified.

## Figures and Tables

**Table 1:** Summary of the mechanical properties of the untreated and vacuum heat-treated bamboo/epoxy composites.

Treatment Condition	Tensile Strength (MPa)	Tensile Modulus (GPa)	Flexural Strength (MPa)	Flexural Modulus (GPa)	Impact Strength (kJ m^−2^)	Shear Strength (MPa)
Untreated	116.64 ± 8.96	16.00 ± 0.23	169.58 ± 3.49	16.54 ± 0.54	20.36 ± 4.28	11.80 ± 0.87
140 °C × 4 h	159.76 ± 7.11	17.92 ± 1.01	208.27 ± 3.82	15.69 ± 1.03	22.43 ± 3.25	12.16 ± 0.54
140 °C × 6 h	168.91 ± 10.53	18.33 ± 0.43	208.59 ± 8.16	15.39 ± 0.65	26.79 ± 1.26	14.26 ± 2.73
160 °C × 4 h	174.49 ± 4.38	16.98 ± 0.51	219.55 ± 14.19	14.60 ± 0.49	27.55 ± 5.62	16.75 ± 0.92
160 °C × 6 h	163.47 ± 3.40	18.14 ± 0.53	211.86 ± 9.27	14.74 ± 0.34	24.33 ± 6.80	12.59 ± 0.97
180 °C × 4 h	170.13 ± 9.64	17.31 ± 0.95	198.63 ± 4.14	14.96 ± 0.88	19.84 ± 4.65	11.64 ± 0.76
180 °C × 6 h	151.81 ± 7.01	16.50 ± 0.19	194.66 ± 10.34	13.88 ± 0.50	14.05 ± 4.00	11.05 ± 1.55

**Table 2:** Mass changes of bamboo strips after vacuum heat treatment.

Treatment Condition	Before (g)	After (g)	Mass Change (%)
140 °C × 4 h	11.73	11.05	−5.80
140 °C × 6 h	10.38	9.27	−10.69
160 °C × 4 h	9.66	8.26	−14.49
160 °C × 6 h	9.86	7.94	−19.47
180 °C × 4 h	9.99	7.54	−24.52
180 °C × 6 h	10.02	7.39	−26.25

## Data Availability

The original contributions presented in this study are included in the article. Further inquiries can be directed to the corresponding authors.
